# Copeptin relates to a fatty liver and measures of obesity in a South African population with mixed ethnicities

**DOI:** 10.1007/s12020-019-01977-y

**Published:** 2019-06-22

**Authors:** Sofia Enhörning, Léone Malan

**Affiliations:** 1 Department of Clinical Science, Lund University, Skåne University Hospital, SE 214 28 Malmö, Sweden; 2 Department of Endocrinology, Skåne University Hospital, SE 221 85 Lund, Sweden; 3 Hypertension in Africa Research Team (HART), Centre of Excellence, North-West University, Private Bag x6001, Potchefstroom, 2520 South Africa

**Keywords:** Vasopressin, NAFLD, Triglycerides, Gamma glutamyl transferase

## Abstract

**Purpose:**

Elevated copeptin, a vasopressin marker, is linked to metabolic disease, and obese rats with low-vasopressin concentration had a decreased risk of liver steatosis. We here investigated the association between copeptin and nonalcoholic fatty liver disease (NAFLD) and possible differences in copeptin concentration between ethnicities.

**Methods:**

In this cross-sectional study of 361 South Africans (*n* = 172 African black, 189 = Caucasian) with a mean age of 45 years and 45% men, plasma copeptin was measured and associated with NAFLD according to a validated fatty liver index accounting for measures of BMI, waist, triglycerides, and gamma-glutamyltransferase.

**Results:**

There was no significant difference in copeptin concentrations between ethnicities after age and gender adjustment (*p* = 0.24). Increasing copeptin tertile levels were significantly associated with obesity, overweight, and abdominal obesity, respectively, after multivariate adjustment for age, gender, ethnicity, and high HOMA-IR (*p* = 0.02 for all). Individuals in the second and third copeptin tertile had an increased odds (95% CI) of NAFLD of 1.77 (1.04–3.02) and 2.97 (1.74–5.06), respectively, compared to the bottom tertile (*p* < 0.001). The association between increasing copeptin tertile and NAFLD remained significant after adjustment for age, gender, ethnicity, high HOMA-IR, self-reported current alcohol intake, and statin treatment (*p* = 0.01).

**Conclusions:**

Elevated plasma copeptin is independently associated with NAFLD in a population with mixed ethnicities, pointing at the pharmacologically modifiable vasopressin system as a new mechanism behind NAFLD.

## Introduction

Nonalcoholic fatty liver disease (NAFLD) is the most frequent liver disease in Western countries [1], with a prevalence that has increased in parallel with increasing burden of metabolic diseases [2]. The condition, which has a multifactorial pathophysiological background, is defined as either fat accumulation in the liver with more than 5% of hepatocytes containing visible intracellular triglycerides, or steatosis affecting at least 5% of the liver volume or weight, in the absence of a secondary cause such as alcohol or drugs [3]. The validated Bedogni fatty liver index is a surrogate marker for fatty liver which is calculated using body mass index, waist circumference, triglycerides, and gamma-glutamyltransferase (GGT) [1].

There is a close association between NAFLD and type 2 diabetes, obesity and dyslipidemia, respectively, and NAFLD has been called the hepatic component of the metabolic syndrome as its presence and severity is associated with components of the syndrome [4]. Furthermore, NAFLD is an independent risk factor for development of type 2 diabetes [5], and is suggested to play a pathophysiological role in the development of atherosclerosis [6].

We previously showed in population-based studies that high copeptin, a stable plasma marker of vasopressin (VP), is independently associated with all components of the metabolic syndrome [7–9], increased risk of developing type 2 diabetes and overweight [7, 8] as well as increased risk of heart disease and cardiovascular mortality [10–12]. In the liver, VP stimulates hepatic production of triglycerides [13]. Accordingly, recent data from 60 obese patients showed that those with biopsy-proven NAFLD had significantly higher copeptin concentrations than those without NAFLD. Furthermore, experimental work show that obese rats with water induced reduction of VP concentration in plasma have decreased liver steatosis compared with control obese rats [14]. It is not yet known whether the link between high levels of circulating VP (copeptin) and metabolic disturbances is causal, however, both an experimental study in rats [14] and a human Mendelian randomization study [15] point at a causal relationship.

Obesity, dyslipidemia, insulin resistance, type 2 diabetes, and heart disease have thus all been shown to associate with both fatty liver disease and elevated VP (copeptin) concentration. This study aims at for the first time investigating the links between copeptin concentration and fatty liver disease, as knowledge of the underlying causes of NAFLD is a prerequisite for the identification of more specific lifestyle and pharmacological treatment. Furthermore, as the risk of developing NAFLD is suggested to differ by ethnicity [16, 17], this study aims at for the first time investigating copeptin concentration as well as links between fatty liver disease and plasma copeptin concentrations in different ethnic groups.

## Materials and methods

### Study design and participants

The population of this study is derived from the sympathetic activity and ambulatory blood pressure in Africans (SABPA) study, a target population study with a total of 409 teachers that was conducted from 2008 to 2009. The reason for this selection was to obtain a homogenous sample from a similar socioeconomic class and is well-described elsewhere [18]. Out of the 409 participants, 200 were Africans (101 males and 99 females) and 209 were Caucasians (101 males and 108 females). Participants were between 20 and 62 years of age. The original exclusion criteria for the SABPA study were pregnancy, lactation, use of alpha- or beta-blockers, psychotropic substance abuse, vaccination or blood donation within 3 months prior to participation and a body temperature above 37.5 °C. Furthermore, participants who tested positive for the human immunodeficiency virus were excluded (*n* = 19). Out of the remaining 390 subjects, we were able to determine baseline plasma copeptin in 361 individuals, which is the final study sample.

### Clinical examination

Clinical assessments were conducted over a 2-day period. On the first morning, 24 h ambulatory blood pressure monitoring was conducted during a working day. It was programmed to measure blood pressure at intervals of 30 min between 08:00 and 22:00 h and intervals of 60 min between 22:00 and 06:00 (Cardiotens CE120^®^, Meditech, Budapest, Hungary). Participants were transported at 16:30 h to the Metabolic Unit Research Facility of the North-West University. After receiving a standardized dinner, participants were encouraged to go to bed at around 22:00 h and were woken up at 05:45 h the following morning to undergo a battery of clinical assessments. Registered nurses collected fasting venous blood samples after participants were in a semi-recumbent position for at least 30 min. Information about prescribed medications and medical history were obtained. Anthropometric measurements were taken in triplicate by registered anthropometrists.

### Laboratory measurements

Sodium fluoride, plasma, and serum samples from fasting blood were stored at −80 °C before the analyses. Fasting sodium fluoride (glucose) and serum samples for high density lipoprotein (HDL) cholesterol, triglycerides, GGT, and high-sensitivity C-reactive protein (CRP) were analyzed using two sequential multiple analyzers (Konelab 20i; Thermo Scientific, Vantaa, Finland; Unicel DXC 800—Beckman and Coulter^®^, Germany). Fasting serum insulin was measured using an immunoassay (Elecsys 2010, Roche, Basel, Switzerland). Whole blood was used to measure HbA1c using a turbidometric inhibition immunoassay (Cobas Integra 400, Roche, Switzerland).

Fasting plasma copeptin concentration was measured using a KRYPTOR Compact Plus device and commercially available chemiluminescence sandwich immunoassay copeptin ProAVP kit with coated tubes from samples stored at −80 °C (Thermo Scientific BRAHMS Copeptin proAVP KRYPTOR).

### Calculation and definition of metabolic variables

The validated Bedogni fatty liver index, an algorithm based on body mass index (BMI), waist circumference, triglycerides and GGT was calculated. The index varies between 0 and 100 and an index ≥60 is considered to rule in liver steatosis [19, 20]. Thus, a fatty liver index ≥60 was defined as NAFLD.

Overweight was defined as BMI ≥ 25 and obesity was defined as BMI ≥ 30 [21]. Abdominal obesity was defined as waist circumference >102 cm in men and >88 cm in women [22]. Twenty-four hour hypertension was defined as ≥130 and/or 80 mmHg.

HOMA-IR was calculated using the following algorithm: fasting glucose (mmol/L) * fasting insulin (mU/L)/22.5 [23]. To classify insulin resistance, we used high HOMA-IR >3.8 and top quartile of insulin concentration.

Low HDL was defined as a concentration <1.0 mmol/L for men and <1.3 mmol/L for women [22].

### Statistical analyses

Statistical analyses were performed using SPSS statistical software (version 24.0). Group-wise differences in continuous variables were tested with the Student's *t* test and reported as mean ± SD if normally distributed and with the Mann–Whitney *U* test and reported as medians and interquartile ranges if not normally distributed. Variables that were not normally distributed were transformed with the natural logarithm when analyzed as continuous variables. Frequency differences in dichotomous variables were evaluated using chi-square test. As copeptin is known to be significantly higher in men than in women, we used sex-specific pooled tertiles of copeptin throughout the manuscript.

Univariate and multivariate adjusted logistic regression models were used to test the relationship between tertiles of copeptin and binary outcome variables, whereas univariate and multivariate adjusted linear regression models were used to test the relationship between tertiles of copeptin and continuous outcome variables. A two-sided *p<* 0.05 was considered statistically significant. Interaction was tested by introducing interaction terms on top of all other covariates in the logistic regression model to test for interaction on prevalence of high fatty liver index (copeptin tertile × insulin concentration and copeptin × ethnicity, respectively).

## Results

The present study consisted of a South African population. Out of 409 participants, complete data was available in the majority of participants (*n* = 361) who had a mean age 45 years, 45% were men and 48% were African (Table 1). There was no significant difference in copeptin concentration between ethnicities (African/Caucasian) after age and gender adjustment (*p* = 0.24).

**Table 1:** Population description (*N* = 361)

	All participants	African ethnicity, *N* = 172	Caucasian ethnicity, *N* = 189	*p* Value, African vs. Caucasian
Age (y)	44.8 (9.7)	44.4 (8.4)	45.1 (10.8)	0.49
Sex, *n* (%) men	163 (45.2)	82 (47.7)	81 (42.9)	0.36
Copeptin (pmol/L)^a^	4.45 (3.18; 7.27)	4.70 (3.40; 7.52)	4.20 (3.01; 6.83)	0.07
BMI (kg/m^2^)	28.9 (6.7)	30.4 (7.0)	27.5 (6.0)	<0.001
Obesity, *n* (%)	133 (36.8)	85 (49.4)	48 (25.4)	<0.001
Overweight, *n* (%)	247 (68.4)	130 (75.6)	117 (61.9)	0.005
Waist circumference (cm)	92.9 (15.8)	93.8 (15.5)	92.1 (16.0)	0.32
Abdominal obesity (%)	154 (42.7)	83 (48.3)	71 (37.6)	0.04
Fatty liver index^a^	49 (14; 81)	64 (31; 87)	31 (8;72)	<0.001
High fatty Liver Index >60, *n* (%)	152 (42.1)	91 (52.9)	61 (32.3)	<0.001
24 h Hypertension, *n* (%)	115 (31.9)	82 (47.7)	33 (17.5)	<0.001
% HbA1c^a^	5.6 (5.3; 5.9)	5.7 (5.5; 6.2)	5.4 (5.2; 5.7)	<0.001
Glucose (mmol/L)^a^	5.8 (5.4; 6.3)	5.9 (5.4; 6.4)	5.8 (5.5; 6.2)	0.23
Insulin (mU/L)^a^	10.89 (7.46; 15.76)	12.32 (8.48; 17.11)	9.52 (6.65; 14.51)	<0.001
HOMA-IR^a^	2.9 (1.9; 4.5)	3.3 (2.1; 5.5)	2.5 (1.7; 3.8)	<0.001
HOMA-IR >3.8, *n* (%)	119 (33.0)	73 (42.4)	46 (24.3)	<0.001
Triglycerides (mg/dl)^a^	90.7 (63.7; 134.2)	95.7 (66.4; 132.8)	83.2 (61.1; 124.3)	0.04
HDL (mmol/L)	1.19 (0.38)	1.15 (0.33)	1.22 (0.41)	<0.05
Low HDL, *n* (%)	196 (54.3)	100 (58.1)	96 (50.8)	0.16
C-reactive protein^a^	2.8 (1.0; 6.6)	5.1 (2.1; 9.9)	1.6 (1.0–3.8)	<0.001
Gamma glutamyltransferase (U/L)^a^	27.0 (15.4; 47.4)	40.1 (27.5; 67.0)	17.0 (12.0; 28.0)	<0.001

Data are given as mean ± SD if nothing else specified

^a^Expressed as median (25th; 75th percentile)

Increasing copeptin tertile was after adjustment for age, gender, and ethnicity significantly associated with elevated HbA1c, insulin, HOMA-IR, BMI, overweight, obesity, abdominal obesity, waist circumference, and decreased HDL, whereas the association between copeptin and fasting glucose and GGT, respectively, did not remain significant after multivariate adjustment (Table 2). The association between increasing copeptin tertile and obesity, overweight and abdominal obesity, respectively, remained after additional adjustment for high HOMA-IR (*p* = 0.02 for all). Furthermore, increasing copeptin tertile had an increased odds (95% CI) of NAFLD (according to the Bedogni high fatty liver index) of 1.77 (1.04–3.02) and 2.97 (1.74–5.06), respectively, compared to the bottom tertile (*p* < 0.001). The association remained significant after adjustment for age, gender, and ethnicity (Table 2) as well as after additional adjustments for high HOMA-IR, self-reported current alcohol intake and statin treatment (*p* = 0.01). The effect of all variables in the multivariate adjusted analyses (i.e., copeptin tertile, gender, age, ethnicity, and high HOMA-IR) on the occurrence of measures of obesity and NAFLD is shown in Table 3.

**Table 2:** Glucometabolic factors in sex-specific tertiles of copeptin

	Copeptin tertile 1 median (min–max) copeptin: (4.05 (1.19–5.05) in men, 2.30 (1.08–3.02) in women, pmol/l) *N* = 120, 45% African ethnicity	Copeptin tertile 2 median (min–max) copeptin: (6.47 (5.10–8.13) in men, 3.60 (3.03–4.23) in women, pmol/l) *N* = 121, 41% African ethnicity	Copeptin tertile 3 median (min–max) copeptin: (10.98 (8.17–23.73) in men, 5.50 (4.26–62.4) in women, pmol/l) *N* = 120, 57% African ethnicity	*P* value univariate	*P* value multivariate^a^
Glucose (mmol/L)	5.7 (5.4; 6.2)	5.8 (5.4; 6.2)	5.9 (5.5; 6.5)	0.01	0.09
% HbA1c	5.5 (5.3; 5.9)	5.5 (5.3; 5.7)	5.7 (5.4; 6.1)	0.002	0.03
Insulin (mU/L)	8.7 (6.3; 14.8)	10.7 (7.6; 14.6)	13.1 (9.4; 19.5)	<0.001	<0.001
HOMA-IR	2.3 (1.6; 3.8)	2.7 (1.9; 4.1)	3.5 (2.3; 5.7)	<0.001	<0.001
HOMA-IR >3.8 (%)	25.0	28.1	45.8	0.001	0.003
Triglycerides (mmol/L)	0.95 (0.69; 1.38)	0.94 (0.70; 1.56)	1.09 (0.78; 1.45)	0.16	0.35
HDL (mmol/L)^b^	1.24 (0.37)	1.21 (0.42)	1.12 (0.32)	0.01	0.003
Low HDL (%)	48.3	50.4	64.2	0.01	0.009
C-reactive protein (mg/L)	2.42 (0.99; 5.68)	2.29 (0.99; 5.48)	3.77 (1.40; 8.32)	0.02	0.13
BMI (kg/m^2^)^b^	26.7 (5.4)	29.0 (6.8)	31.0 (7.1)	<0.001	<0.001
Overweight (%)	57.5	67.8	80.0	<0.001	0.001
Obesity (%)	24.2	38.8	47.5	<0.001	0.001
Waist circumference (cm)^b^	87.5 (13.0)	92.9 (15.8)	98.2 (16.6)	<0.001	<0.001
Abdominal obesity (%)	31.7	40.5	55.8	<0.001	0.001
Gamma Glutamyltransferase (U/L)	26.4 (13.0; 41.8)	22.8 (15.0; 50.2)	30.1 (21.7; 56.3)	0.03	0.19
NAFLD (%)	29.2	42.1	55.0	<0.001	<0.001

Data expressed as median (25th; 75th percentile) if nothing else specified

*P* value in linear regression

^a^Multivariate linear regression adjusted for age, sex, and ethnicity

^b^Expressed as mean (standard deviation)

**Table 3:** The effect of copeptin tertile, gender, age, ethnicity, and high HOMA-IR on occurrence of measures of obesity and NAFLD

	Variables in the analysis	Comparison	Odds ratio (95% CI)	*p* Value multivariate
*Obesity*				
	Copeptin tertile	2 vs. 1	2.26 (1.21–4.25)	0.01
		3 vs. 1	2.08 (1.11–3.91)	0.02
	Gender	Female vs. Male	2.25 (1.32–3.83)	0.003
	Age		1.00 (0.97–1.03)	0.99
	Ethnicity	Caucasian vs. African	0.41 (0.25–0.68)	<0.001
	HOMA-IR	High (>3.8) vs. low	7.05 (4.08–12.18)	<0.001
*Overweight*				
	Copeptin tertile	2 vs. 1	1.65 (0.92–2.95)	0.09
		3 vs. 1	2.03 (1.07–3.86)	0.03
	Gender	Female vs. Male	1.08 (0.65–1.81)	0.76
	Age		1.02 (0.996–1.05)	0.10
	Ethnicity	Caucasian vs. African	0.70 (0.41–1.17)	0.17
	HOMA-IR	High (>3.8) vs. low	16.38 (6.37–42.14)	<0.001
*Abdominal obesity*				
	Copeptin tertile	2 vs. 1	1.57 (0.86–2.88)	0.14
		3 vs. 1	2.00 (1.08–3.69)	0.03
	Gender	Female vs. Male	2.99 (1.73–5.16)	<0.001
	Age		1.02 (0.99–1.05)	0.18
	Ethnicity	Caucasian vs. African	0.91 (0.55–1.49)	0.70
	HOMA-IR	High (>3.8) vs. low	10.44 (5.84–18.69)	<0.001
*NAFLD*				
	Copeptin tertile	2 vs. 1	2.18 (1.13–4.22)	0.02
		3 vs. 1	2.35 (1.21–4.56)	0.01
	Gender	Female vs Male	0.54 (0.32–0.92)	0.02
	Age		1.00 (0.97–1.03)	0.89
	Ethnicity	Caucasian vs. African	0.54 (0.32–0.92)	0.02
	HOMA-IR	High (>3.8) vs. low	13.70 (7.66–24.49)	<0.001

As hyperinsulinemia is common in subjects with fatty liver and thus expected to be a marker of fatty liver in the general population, and as copeptin is known to be associated with hyperinsulinemia at a population level [7], we additionally used another measure of insulin resistance than high HOMA-IR, i.e., top quartile of insulin concentration, when analyzing the association between NAFLD and increasing copeptin tertile, but did not find the multivariate adjusted association to be affected (*p* < 0.001). Furthermore, we investigated if there were any significant interaction between insulin concentration and copeptin tertile on the prevalence of high fatty liver index, which we did not find (*p* = 0.94).

Finally, there was no significant interaction between ethnicity and copeptin tertile on prevalence of high fatty liver index (*p* = 0.51).

## Discussion

In this study, we show for the first time in a population based study a strong association between elevated copeptin and NAFLD. Furthermore, to our knowledge this is the first population based study designed to investigate levels of copeptin between different ethnicities. We did not find any significant difference in copeptin concentration between ethnicities. Neither did we find that the association between copeptin and NAFLD was affected after adjustment for ethnicity, which underlines that our data seems to be generalizable to a mixed population.

Our finding that high circulating concentrations of the VP marker copeptin was linked to higher occurrence of NAFLD is in line with our previous findings that obese rats with water induced reduction of VP concentration have a lower risk of liver steatosis compared with control rats [14] and that obese patients with biopsy-proven NAFLD had significantly higher copeptin concentrations than both obese patients without NAFLD and nonobese individuals [24]. As fatty liver is known to be the liver component of the metabolic syndrome, the NAFLD finding was not surprisingly paralleled with associations between copeptin and measures of obesity and impaired glucose tolerance, findings which are completely corresponding to our previous studies in which we established elevated copeptin as an independent risk factor involved in development of diabetes and the metabolic syndrome [7–9]. By now showing that copeptin, independently of age, gender, and ethnicity, was positively associated to elevated insulin, BMI, and waist circumference, and negatively associated to HDL, we are not only able to replicate our previous findings [7, 9], but these data also add to the current knowledge by showing that the finding are generalizable to a population with mixed ethnicities. Furthermore, we now found a completely novel association between copeptin and elevated HbA1c.

Metabolic effects from altered VP secretion (measured as copeptin) can thus be expected, and previous experiments in rats [14] and a human mendelian randomization study [15] suggests causality between elevated VP and metabolic dysregulation. There are several potential mechanistical explanations behind this link; VP mediates gluconeogenesis and glycogenolysis through VP 1a receptors which are widely expressed in the hepatic tissue [25, 26]. Furthermore, VP stimulates secretion of either glucagon or insulin through VP 1b receptors in the pancreas [27]. Moreover, VP mediates ACTH release through VP 1b receptors in the anterior pituitary, which thus elevates glucocorticoid levels in plasma upon stressful stimuli [28]. This VP-induced ACTH release has been reported to be resistant to glucocorticoid feedback in contrast to the corticotropin-releasing hormone-induced ACTH release [29]. It may suggest that excessive VP release, induced by, for example, relative dehydration, stress, or genetic predisposition, overstimulates the HPA axis and elevates glucocorticoid levels, leading to development of a mild Cushing-like phenotype with overweight, obesity, and insulin resistance [30].

A study on isolated rat hepatocytes showed that VP stimulates hepatic production of triglycerides [13]. Furthermore, knock-out mice lacking expression of the VP 1a receptors have increased lipolysis compared to wild-type mice [31], and we previously showed that genetic variation in the human VP 1a receptor was associated with altered BMI and a fenotype resembling the knock-out mice, including elevated glucose and low triglycerides [32, 33]. Hypothetically, elevated VP (measured as copeptin) may thus play a direct role in intrahepatic fat accumulation by stimulating the VP 1a receptors expressed in the liver and in this way modulate lipid metabolism toward anti-lipolysis.

According to the literature, there are known differences in incidence and severity of liver steatosis between different ethnic groups. Most notably, black ethnicity is known to be protective, whereas Hispanic groups are at higher risk [16]. However, we did not find any significant interaction between ethnicity and copeptin tertile on prevalence of NAFLD (*p* = 0.51), and the number of individuals with NAFLD was significantly higher among Africans (60%) than among Caucasians (32%) in our population. As described previously, the African and Caucasian sample was well matched for age and sex distribution. However, in general, the Africans demonstrated more metabolic and cardiovascular risk factors, including higher ambulatory systolic and diastolic blood pressure, triglycerides, BMI, CRP, and a higher prevalence of metabolic syndrome [34]. Thus, the much higher prevalence of NAFLD among the Africans is in line with previously published data on the differences in metabolic risk between different ethnicity in the current population.

As there are not any clinically accepted definitions for insulin resistance, we choose to use two different classifications of insulin resistance, high HOMA-IR >3.8 and top quartile of insulin concentration. The latter definition was chosen as we previously used this definition when investigating links between copeptin and insulin sensitivity in a Swedish middle-aged population [7], whereas a HOMA-IR >3.8 was chosen as this cut-off level was previously shown to have very good sensitivity and specificity in a Hispanic population with very similar prevalence of insulin resistance as in our population (39% insulin resistance in the Hispanic population, 34% in our population if using the HOMA-IR >3.8 definition) [35].

The VP system has been thoroughly studied in relation to the development of liver cirrhosis. Baroreceptor-induced secretion of VP is seen in liver cirrhosis, and copeptin has been shown to be associated with clinical decompensation, development of complications, and mortality of liver cirrhosis [36]. Furthermore, VP receptor antagonism has been suggested for the treatment of hyponatremia in cirrhosis [37]. In this study, none of the participants had a liver cirrhosis diagnosis.

### Limitations

Most importantly, the study has a cross-sectional design, and no conclusions can thus be drawn on the cause–effect relationship of the found associations.

Ethanol intake is suggested to play a role in development of fatty liver [38]. We additionally adjusted for self-reported current alcohol intake in the model analyzing the impact of copeptin on prevalence of NAFLD which did not affect the association. However, the alcohol intake variable was a self-reported questionnaire-based variable with only current consumption, yes or no, as alternatives. As previously shown, the Africans reported a lower use of alcohol, but both African alcohol users and nonusers demonstrated higher levels of GGT than the Caucasians [34]. The elevated GGT may be a reflection of higher prevalence of NAFLD in the African part of the population, but may also reflect higher alcohol intake among Africans than among Caucasians.

The limitations of the study underlines the importance of further investigating the association between copeptin and fatty liver disease, preferably using more sensitive methods to define NAFLD such as ultrasonography or computerized tomography of the liver.

In conclusion, our data show for the first time a link between altered regulation of the VP system measured as elevated circulating copeptin concentration, and presence of NAFLD in the population, suggesting an completely new and potentially modifiable risk factor behind fatty liver disease.

## References

[1] Bedogni G, Nobili V, Tiribelli C (2014). Epidemiology of fatty liver: an update. World J. Gastroenterol..

[2] Younossi ZM, Koenig AB, Abdelatif D, Fazel Y, Henry L, Wymer M (2016). Global epidemiology of nonalcoholic fatty liver disease—meta-analytic assessment of prevalence, incidence, and outcomes. Hepatology.

[3] Abd El-Kader SM, El-Den Ashmawy EM (2015). Non-alcoholic fatty liver disease: the diagnosis and management. World J. Hepatol..

[4] Marchesini G, Bugianesi E, Forlani G, Cerrelli F, Lenzi M, Manini R, Natale S, Vanni E, Villanova N, Melchionda N, Rizzetto M (2003). Nonalcoholic fatty liver, steatohepatitis, and the metabolic syndrome. Hepatology.

[5] Sung KC, Wild SH, Byrne CD (2013). Resolution of fatty liver and risk of incident diabetes. J. Clin. Endocrinol. Metab..

[6] Sinn DH, Kang D, Chang Y, Ryu S, Gu S, Kim H, Seong D, Cho SJ, Yi BK, Park HD, Paik SW, Song YB, Lazo M, Lima JA, Guallar E, Cho J, Gwak GY (2017). Non-alcoholic fatty liver disease and progression of coronary artery calcium score: a retrospective cohort study. Gut.

[7] Enhorning S, Struck J, Wirfalt E, Hedblad B, Morgenthaler NG, Melander O (2011). Plasma copeptin, a unifying factor behind the metabolic syndrome. J. Clin. Endocrinol. Metab..

[8] Enhorning S, Wang TJ, Nilsson PM, Almgren P, Hedblad B, Berglund G, Struck J, Morgenthaler NG, Bergmann A, Lindholm E, Groop L, Lyssenko V, Orho-Melander M, Newton-Cheh C, Melander O (2010). Plasma copeptin and the risk of diabetes mellitus. Circulation.

[9] Enhorning S, Bankir L, Bouby N, Struck J, Hedblad B, Persson M, Morgenthaler NG, Nilsson PM, Melander O (2013). Copeptin, a marker of vasopressin, in abdominal obesity, diabetes and microalbuminuria: the prospective Malmo Diet and Cancer Study cardiovascular cohort. Int J. Obes..

[10] Enhorning S, Hedblad B, Nilsson PM, Engstrom G, Melander O (2015). Copeptin is an independent predictor of diabetic heart disease and death. Am. Heart J..

[11] Tasevska I, Enhorning S, Persson M, Nilsson PM, Melander O (2016). Copeptin predicts coronary artery disease cardiovascular and total mortality. Heart.

[12] Velho G, El Boustany R, Lefevre G, Mohammedi K, Fumeron F, Potier L, Bankir L, Bouby N, Hadjadj S, Marre M, Roussel R (2016). Plasma copeptin, kidney outcomes, ischemic heart disease, and all-cause mortality in people with long-standing type 1 diabetes. Diabetes Care..

[13] Pollard AD, Brindley DN (1984). Effects of vasopressin and corticosterone on fatty acid metabolism and on the activities of glycerol phosphate acyltransferase and phosphatidate phosphohydrolase in rat hepatocytes. Biochem J..

[14] Taveau C, Chollet C, Waeckel L, Desposito D, Bichet DG, Arthus MF, Magnan C, Philippe E, Paradis V, Foufelle F, Hainault I, Enhorning S, Velho G, Roussel R, Bankir L, Melander O, Bouby N (2015). Vasopressin and hydration play a major role in the development of glucose intolerance and hepatic steatosis in obese rats. Diabetologia.

[15] R. Roussel, R. El Boustany, N. Bouby, L. Potier, F. Fumeron, K. Mohammedi, B. Balkau, J. Tichet, L. Bankir, M. Marre, G. Velho, Plasma copeptin, AVP gene variants, and incidence of type 2 diabetes in a cohort from the community. J Clin Endocrinol Metab. **101**, 2432–2439 (2016) 10.1210/jc.2016-1113 PMC4891798 27049477

[16] Bambha K, Belt P, Abraham M, Wilson LA, Pabst M, Ferrell L, Unalp-Arida A, Bass N (2012). Nonalcoholic Steatohepatitis Clinical Research Network Research G. Ethnicity and nonalcoholic fatty liver disease. Hepatology.

[17] Rich NE, Oji S, Mufti AR, Browning JD, Parikh ND, Odewole M, Mayo H, Singal AG (2018). Racial and ethnic disparities in nonalcoholic fatty liver disease prevalence, severity, and outcomes in the united states: a systematic review and meta-analysis. Clin. Gastroenterol. Hepatol..

[18] Malan L, Hamer M, Frasure-Smith N, Steyn HS, Malan NT (2015). Cohort profile: sympathetic activity and ambulatory blood pressure in Africans (SABPA) prospective cohort study. Int J. Epidemiol..

[19] Bedogni G, Bellentani S, Miglioli L, Masutti F, Passalacqua M, Castiglione A, Tiribelli C (2006). The fatty liver index: a simple and accurate predictor of hepatic steatosis in the general population. BMC Gastroenterol..

[20] Cuthbertson DJ, Weickert MO, Lythgoe D, Sprung VS, Dobson R, Shoajee-Moradie F, Umpleby M, Pfeiffer AF, Thomas EL, Bell JD, Jones H, Kemp GJ (2014). External validation of the fatty liver index and lipid accumulation product indices, using 1H-magnetic resonance spectroscopy, to identify hepatic steatosis in healthy controls and obese, insulin-resistant individuals. Eur. J. Endocrinol..

[21] Haslam DW, James WP (2005). Obesity. Lancet.

[22] Expert Panel on Detection, Evaluation, and Treatment of High Blood Cholesterol in Adults (2001). Executive Summary of The Third Report of The National Cholesterol Education Program (NCEP) Expert Panel on Detection, Evaluation, And Treatment of High Blood Cholesterol In Adults (Adult Treatment Panel III). J. Am. Med. Assoc..

[23] Matthews DR, Hosker JP, Rudenski AS, Naylor BA, Treacher DF, Turner RC (1985). Homeostasis model assessment: insulin resistance and beta-cell function from fasting plasma glucose and insulin concentrations in man. Diabetologia.

[24] Barchetta I, Enhorning S, Cimini FA, Capoccia D, Chiappetta C, Di Cristofano C, Silecchia G, Leonetti F, Melander O, Cavallo MG (2019). Elevated plasma copeptin levels identify the presence and severity of non-alcoholic fatty liver disease in obesity. BMC Med..

[25] Whitton PD, Rodrigues LM, Hems DA (1978). Stimulation by vasopressin, angiotensin and oxytocin of gluconeogenesis in hepatocyte suspensions. Biochem J..

[26] Keppens S, de Wulf H (1979). The nature of the hepatic receptors involved in vasopressin-induced glycogenolysis. Biochim Biophys. Acta.

[27] Abu-Basha EA, Yibchok-Anun S, Hsu WH (2002). Glucose dependency of arginine vasopressin-induced insulin and glucagon release from the perfused rat pancreas. Metabolism.

[28] Tanoue A, Ito S, Honda K, Oshikawa S, Kitagawa Y, Koshimizu TA, Mori T, Tsujimoto G (2004). The vasopressin V1b receptor critically regulates hypothalamic-pituitary-adrenal axis activity under both stress and resting conditions. J. Clin. Invest..

[29] Rabadan-Diehl C, Aguilera G (1998). Glucocorticoids increase vasopressin V1b receptor coupling to phospholipase C. Endocrinology.

[30] Anagnostis P, Athyros VG, Tziomalos K, Karagiannis A, Mikhailidis DP (2009). Clinical review: the pathogenetic role of cortisol in the metabolic syndrome: a hypothesis. J. Clin. Endocrinol. Metab..

[31] Hiroyama M, Aoyagi T, Fujiwara Y, Birumachi J, Shigematsu Y, Kiwaki K, Tasaki R, Endo F, Tanoue A (2007). Hypermetabolism of fat in V1a vasopressin receptor knockout mice. Mol. Endocrinol..

[32] Masuki S, Mori M, Tabara Y, Miki T, Sakurai A, Morikawa M, Miyagawa K, Higuchi K, Nose H (2010). Vasopressin V1a receptor polymorphism and interval walking training effects in middle-aged and older people. Hypertension.

[33] Enhorning S, Leosdottir M, Wallstrom P, Gullberg B, Berglund G, Wirfalt E, Melander O (2009). Relation between human vasopressin 1a gene variance, fat intake, and diabetes. Am. J. Clin. Nutr..

[34] Hamer M, Malan L, Schutte AE, Huisman HW, van Rooyen JM, Schutte R, Fourie CM, Malan NT, Seedat YK (2011). Conventional and behavioral risk factors explain differences in sub-clinical vascular disease between black and Caucasian South Africans: the SABPA study. Atherosclerosis.

[35] Qu HQ, Li Q, Rentfro AR, Fisher-Hoch SP, McCormick JB (2011). The definition of insulin resistance using HOMA-IR for Americans of Mexican descent using machine learning. PLoS ONE.

[36] Sola E, Kerbert AJ, Verspaget HW, Moreira R, Pose E, Ruiz P, Cela R, Morales-Ruiz M, Lopez E, Graupera I (2016). Plasma copeptin as biomarker of disease progression and prognosis in cirrhosis. J. Hepatol..

[37] Gines P, Wong F, Watson H, Milutinovic S, del Arbol LR, Olteanu D (2008). Effects of satavaptan, a selective vasopressin V(2) receptor antagonist, on ascites and serum sodium in cirrhosis with hyponatremia: a randomized trial. Hepatology.

[38] Bellentani S, Tiribelli C (2001). The spectrum of liver disease in the general population: lesson from the Dionysos study. J. Hepatol..

